# Interplay between *AIB1* genotypes and radiotherapy in a Swedish population-based breast cancer cohort

**DOI:** 10.1007/s12672-025-04370-6

**Published:** 2026-01-03

**Authors:** Alexandra Wiberg, Louise Ebbesen, Christopher Godina, Karolin Isaksson, Helena Jernström

**Affiliations:** 1 Division of Oncology, Department of Clinical Sciences Lund, Lund University Cancer Center/Kamprad, Lund University and Skåne University Hospital, Barngatan 4, SE-221 85 Lund, Sweden; 2 Division of Surgery, Department of Clinical Sciences Lund, Lund University Cancer Center, Lund University, SE-22185 Lund, Sweden; 3 Department of Surgery, Skåne University Hospital, Kristianstad, J A Hedlunds väg 5, SE-291 33 Kristianstad, Sweden

**Keywords:** Breast cancer, *AIB1* genotypes, Prognosis, Adjuvant treatment

## Abstract

**Purpose:**

Host factors, including genetic factors, are underutilized in breast cancer treatment selection. This study aimed to investigate *AIB1* genotypes as pharmacogenetic markers for adjuvant breast cancer treatment. The prognostic impact of three functional polymorphisms associated with altered mRNA expression rs6094752 (low expression), rs2230782 (low expression) and rs2076546 (high expression) was studied in different treatment groups.

**Methods:**

*AIB1* genotyping was performed using iPLEX™ on 576 breast cancer patients included 2002 − 2008 in Lund, Sweden, who were followed for up to 15 years. Clinicopathological data was obtained from questionnaires and patient charts. Diplotypes were constructed. Survival analyses were conducted with Kaplan-Meier curves, Log-Rank tests and multivariable Cox regression.

**Results:**

The most common *AIB1* diplotypes were CGA_CGA (61.4%), CGA_CCA (16.4%), and CGA_CGG (12.0%). The remaining diplotypes were classified as ‘Rare’. Any breast cancer event was reported in 144 patients. There were significant interactions between radiotherapy and CGA_CGA (*P*_interaction_=0.033) or CGA_CCA (*P*_interaction_=0.017) on prognosis. In the 226 non-radiotherapy-treated patients, CGA_CGA carriers had the best prognosis, and CGA_CCA carriers the worst prognosis, with two-fold risk of breast cancer events in CGA_CCA compared with CGA_CGA carriers. In the 350 radiotherapy-treated patients, CGA_CGA was not associated with prognosis while CGA_CCA conferred 40% lower event risk. No other significant interactions between *AIB1* diplotypes and chemotherapy, tamoxifen or aromatase inhibitors on prognosis were observed.

**Conclusion:**

*AIB1* genotypes conferred differential prognostic impact depending on adjuvant radiotherapy. If confirmed, *AIB1* merits further evaluation as a putative pharmacogenetic marker to identify patients that benefit the most from radiotherapy.

**Supplementary Information:**

The online version contains supplementary material available at 10.1007/s12672-025-04370-6.

## Introduction

Breast cancer is the most common form of cancer among women worldwide [1]. Although breast cancer survival has improved during the past decades [2, 3], there is still a need for better tailoring of treatment to each patient. Today, treatment decisions are mainly based on tumor-specific properties, such as tumor size, axillary nodal status, histological grade and hormone receptor status and/or molecular subtyping. Besides age, and sometimes body size, host factors are rarely considered. Many breast cancer patients are either overtreated, undertreated or possibly given the wrong treatment [4], leading to increased side-effects and/or decreased treatment efficacy. Therefore, better treatment predictive markers are needed to determine the best treatment for each patient. This includes biomarkers that are patient-specific and not only tumor-specific, such as pharmacogenetic markers. There is a knowledge gap regarding the use of pharmacogenetics in relation to different breast cancer treatment modalities and prognosis. Currently no genes are routinely assessed for all breast cancer patients prior to treatment selection and there are no pharmacogenomic markers for radiotherapy. One interesting marker is the *amplified in breast cancer 1* (*AIB1*) gene that merits investigation as a pharmacogenetic marker for breast cancer treatment.

AIB1 is a member of the p160 steroid receptor coactivator family [5, 6] and is located on chromosome 20q [5]. AIB1 enhances transcription by interacting with nuclear receptors, such as the estrogen and progesterone receptors (ER and PR), in a ligand dependent manner [7]. AIB1 also promotes the transcriptional activity of several different transcription factors [8] and epithelial mesenchymal transition (EMT) drivers [9], mediating cell proliferation, survival, migration and metastasis [10, 11]. Others have shown AIB1 to be associated with more aggressive tumor characteristics in breast cancer, such as human epithelial growth factor receptor-2 (HER2) amplification (HER2^+^) and larger tumor size [9, 12, 13].

The *AIB1* gene is highly polymorphic with multiple single nucleotide polymorphisms (SNPs) [14]. In this study, three functional *AIB1* SNPs are investigated; rs6094752 (652 C > T; C218R), rs2230782 (1758G > C; Q586H) and rs2076546 (2880 A > G; T960T). The rs6094752 and rs2230782 SNPs are non-synonymous and confer a change in protein configuration. The rs2076546 is synonymous and alters the codon but not the amino acid. Both synonymous and non-synonymous SNPs can alter the amount and function of proteins [15, 16]. The rs6094752 minor allele was associated with lower *AIB1* mRNA in cartilage in one study [17]. Similarly, the rs2230782 minor allele has been associated with lower *AIB1* mRNA expression in whole blood [18]. Conversely, the minor allele of rs2076546 has been associated with increased *AIB1* mRNA expression in whole blood [18].

Some studies have previously shown a protective association between rs2230782 and/or rs2076546 and breast cancer risk [19, 20], but not all [21]. *AIB1* genotypes have been associated with an idiosyncratic response of insulin like growth factor 1 (IGF-1) levels to oral contraceptives [22], which could potentially explain the link between *AIB1* genotypes and breast cancer risk. Whether there is also an idiosyncratic response to endocrine treatment is currently unknown.

AIB1 interferes with the endocrine treatment tamoxifen as an ER modulator, though there is no clear consensus whether high AIB1 is a positive [12, 23], neutral [24, 25] or negative [26, 27] marker for tamoxifen response. In addition, high *AIB1* expression in cervical cancer cell lines contributed to radioresistance [28]. Whether *AIB1* genotypes could be used as a pharmacogenetic marker in breast cancer warrants investigation.

We hypothesize that different *AIB1* genotypes may influence prognosis both in endocrine and radiotherapy-treated breast cancer patients. We aimed to evaluate whether *AIB1* genotypes impact prognosis in breast cancer patients with special focus on radiotherapy in all patients and different types of adjuvant endocrine therapy in patients with ER^+^ tumors. Another aim was to identify genes and signaling pathways that differ between high and low *AIB1* expressing tumors in relation to tumor ER status.

## Patients and methods

### Study population

**Fig. 1:**
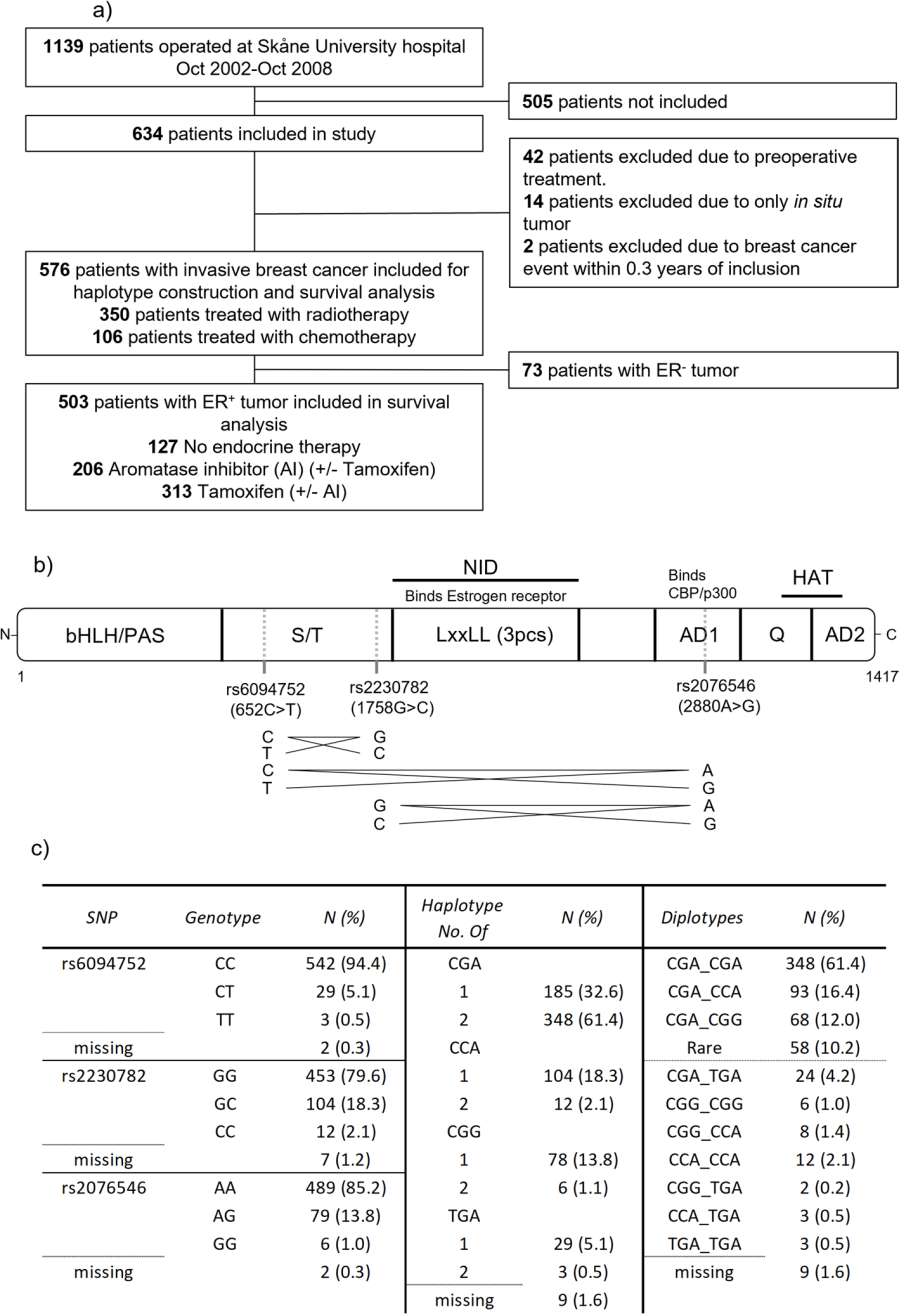
Inclusion flowchart, AIB1 gene, and distribution of AIB1 genotypes. **a** Inclusion flowchart showing number of included and excluded patients. **b** Schematic picture of the *AIB1* gene. The locations of the studied SNPs are indicated, along with a visual representation of linkage disequilibrium between them. **c** The distribution of AIB1 genotypes including SNPs, haplotypes, diplotypes and missing genotypes in the study population. AD: Activation domain; bHLH/PAS: Basic helix–loop–helix–PER–ARNT–SIM; CBP: CREB-binding protein; HAT: Histone acetyltransferase domain; LxxLL: L is leucine, x is any amino acid; NID: Nuclear receptor interaction domain; S/T: Serine/threonine-rich domain; Q: Glutamine rich domain

This study is based on data from the observational study, the breast cancer and blood cohort (BC-blood cohort) at Skåne University Hospital in Lund, which started in October 2002. The current study is based on 634 female patients. Patients with a first breast cancer that were surgically treated between October 2002 and October 2008 were included. Women with a prior breast cancer diagnosis or any other cancer diagnosed within the last 10 years did not meet inclusion criteria. In this study, patients with preoperative treatment, early metastasis, and in situ carcinoma were excluded, leaving 576 included patients. Of these, 503 had ER^+^ tumors (Fig. 1a).

Patients were included at the preoperative visit where they also signed an informed consent. At this visit the patients completed a three-page questionnaire about lifestyle factors (including coffee, smoking and alcohol abstention), reproductive history, medication use and exogenous hormone use. Patients who indicated that they smoked during the last week or smoked during parties were considered smokers. Body measurements, including height and weight, were obtained by the research nurse, who also took blood samples. Body mass index (BMI) was calculated using weight divided by height squared (kg/m^2^). Within two hours after the blood samples were retrieved, they were centrifuged and frozen down to −70 °C.

The follow-up visits with one-page questionnaires took place up to three years postoperatively. Thereafter questionnaires were mailed out biannually and for this study follow-up data collected up until June 30, 2019, were used.

### Tumor characteristics, treatment and breast cancer events

Information about tumor characteristics was retrieved from pathology reports and patient charts. Tumor characteristics that were included in the study were tumor size, histological grade and type, axillary lymph node involvement, ER, PR and HER2 status. Most patients included before November 2005 had missing HER2 status. For these patients, HER2 status was obtained from dual gene protein staining of HER2 on the tissue microarrays and showed 97.7% agreement with pathological assessment [29]. In accordance with the Swedish clinical guidelines, a tumor was considered ER^+^ or PR^+^ if more than 10% was stained nuclear positive. Data on surgery, adjuvant treatments, breast cancer events and death were obtained from patient charts and the Swedish Population Registry. Breast cancer events were defined as any locoregional recurrence, contralateral breast cancer, or distant metastasis. For patients that did not develop any breast cancer event, follow-up time was censored at their last completed questionnaire or death by June 30, 2019.

### Genotyping

To obtain deoxyribonucleic acid (DNA) from buffy coats, the Wizard Genomic DNA purification kit (Promega, Madison, WI, USA) was used in accordance with the manufacturer’s protocol. The *AIB1* SNPs (AIB1_1: rs6094752 (652 C > T; C218R), AIB1_2: rs2230782 (1758G > C; Q586H) and AIB1_3: rs2076546 (2880 A > G; T960T) were analyzed at the Region Skåne Competence Center (RSKC) of Skåne University Hospital in Malmö, Sweden. The software and equipment in the MassARRAY^®^ platform (Sequenom, inc., San Diego, CA, USA) with the reagents included in the iPLEX™ genotyping kit (Sequenom, Inc., San Diego, CA, USA) were used to perform genotype analyses in accordance with the manufacturer’s instructions. About 10% of the samples were run in duplicate and the concordance was 100%. Samples were sent for genotyping in October 2008, and genotypes were therefore available for patients included in the study by October 2008.

### Construction of haplotypes and diplotypes

Cross tabulations were used for each SNP against the other two SNPs in all 576 patients with invasive tumors, irrespective of ER status. Some SNPs were in linkage with each other, and certain combinations did not exist. The results from these crosstabs were then used to construct the four different haplotypes and then build diplotypes (Fig. 1b).

### Statistics

The statistical analyses were performed using SPSS Statistics 28 (IBM, Chicago, IL, USA) and R version 4.3.0. Patient and tumor characteristics as well as adjuvant treatment prior to any breast cancer event were analyzed in relation to *AIB1* SNPs and diplotypes using Chi^2^-test. For each of the three SNPs the homozygous carriers of the common allele vs. carriers of at least one copy of the variant allele were analyzed. There were too few homozygous carriers of the variant alleles for all three SNPs, therefore the hetero- and homozygous variant allele carriers were combined into one group of any variant allele. For diplotypes, the three most common diplotypes were assessed individually and the remaining diplotypes (< 5%) were combined into one ‘Rare’ group.

Several variables were not normally distributed and therefore dichotomized. The dichotomized variables were age at inclusion (≥ 50 years), BMI (≥ 25 kg/m^2^), coffee intake (≥ 2 cups/day), alcohol abstention (yes), invasive tumor size (> 20 mm or skin or muscle involvement), any axillary node involvement (yes) and histological grade (III). Adjuvant treatment was defined as any therapy received before the first breast cancer event or last follow-up, including chemotherapy (ever) and radiotherapy (ever). Endocrine treatment was defined as ever use of tamoxifen or aromatase inhibitors (AIs). The majority of patients received multiple types of adjuvant therapy. There were 11 patients with bilateral tumors, data from the side of the most aggressive cancer are presented in the tables. All but one of the patients with bilateral tumors had the same type of final surgery technique on both sides (breast conserving surgery or mastectomy).

### Statistical analysis

The Chi^2^-test was used to calculate *P*-values when testing associations between clinicopathological factors and SNPs or diplotypes. Univariable and multivariable survival analyses were carried out to investigate whether the diplotypes were associated with breast cancer event incidence by type of adjuvant therapy and if the diplotypes were associated with any breast cancer event, locoregional, contralateral or distant metastasis. The primary endpoint was any breast cancer event. The secondary endpoints were distant metastasis, locoregional recurrence (only in relation to radiotherapy) and contralateral breast cancer (only in relation to tamoxifen treatment). Time to event was calculated as the time between the date of inclusion until each type of breast cancer event. Kaplan-Meier curves were for visualization with corresponding log-rank tests being used to test potential statistical associations. Cox proportional hazard regression was used to calculate crude and adjusted hazard ratios (HR) with 95% CI. The clinically relevant variables included in the model were age at inclusion (≥ 50 years), BMI (≥ 25 kg/m^2^), tumor size (> 20 mm or skin and muscle involvement), any axillary lymph node involvement, histological grade III, ER^+^, HER2^+^, and ever use of adjuvant treatments (chemotherapy, radiotherapy, tamoxifen and AI). For HER2, a third group of ‘unknown’ was added due to the number of patients with missing values (*n* = 62). For the other variables, most patients had complete data and no further imputation was performed. In addition, multiplicative interaction analyses were performed between adjuvant therapies and the *AIB1* SNPs and diplotypes. Interactions with *P* < 0.1 were examined further in stratified subgroups using univariable and multivariable analyses [30]. Sensitivity analyses with final type of surgery added to the model were also performed.

### Power calculations

Power calculations were based on 570 patients, of whom 228 had variant genotypes (not CGA_CGA). The accrual time was 11 years with an additional follow-up of 6 years. Median survival time for patients with the common genotype CGA_CGA was 11 years. True HR of events for variant genotypes relative to common genotypes can be detected for ≤ 0.740 or ≥ 1.394 with 80% power and an alpha of 0.05. Power calculations based on 564 patients where 94 had the CGA_CCA genotype. For these the true HR of events for CGA_CCA relative to other diplotypes can be detected for ≤ 0.675 or ≥ 1.568 with 80% power and an alpha of 0.05 (PS version 3.1.6) [31].

All statistical tests were two-tailed and were regarded as statistically significant if *P* < 0.05. Since this is an explorative study, nominal *P*-values were presented with no adjustment for multiple testing. However, false discovery rate (FDR) was also applied to investigate whether the results held for multiple testing. This study followed the Reporting Recommendations for Tumor Marker Prognostic Studies (REMARK) criteria [32].

### SCAN-B cohort

Differential gene expression (DGE) analysis was conducted in 5326 gene expression profiles of an unilateral breast cancer, each from an unique patient with available follow-up for distant metastasis in The Swedish Cancerome Analysis Network—Breast (SCAN-B: ClinicalTrials.gov ID NCT02306096) cohort [33–36]. Briefly, gene expression data from Illumina Sequencers were processed using the standardized SCAN-B RNA-seq pipeline in BASE with the extension package Reggie, including adapter/quality trimming, HISAT2 alignment, and StringTie quantification [33]. Gene-level values were normalized through SCAN-B’s internal protocol-harmonization procedure, where gene-specific deltas between library protocols (dUTP, NeoPrep, TruSeq) were estimated in clinically balanced subsets and used to transform all samples to a common TruSeq-like baseline [33]. Removal of protocol-related batch effects was verified using SWAMP analyses, which confirmed elimination of technical variation while preserving biological signal [33]. Gene expression values, originally quantified as fragments per kilobase of exon per million mapped reads (FPKM), were offset by + 0.1 and log2-transformed prior to downstream analyses. The ‘Limma-Voom’ package [37] was used to find differentially expressed genes (DEGs) between the highest tertile (T3) and the lowest tertile (T1) of *AIB1* mRNA expression. The criteria used to define DEGs is a false discovery rate (FDR) of ≤ 0.05 and log2 fold change (log2FC) ≥ 1.0 for up-regulated genes and log2FC ≤ −1.0 for down-regulated genes. The package ‘Enhanced Volcanoplot’ was used to visualize the DGE results. Gene set enrichment analysis (GSEA) was performed in ‘clusterprofiler’ [38] to find the statistically significant, concordant gene sets that differed between the highest tertile (T3) and the lowest tertile (T1) of *AIB1* mRNA expression. Gene sets were grouped according to Hallmark Signature annotations [39, 40]. The GSEA results were visualized using ‘clusterprofiler’ [38].

This study was approved by the ethics committee at Lund university (Dnr 75 − 02, Dnr 37 − 08, Dnr 658-09 and amendments). All patients signed written informed consent.

## Results

### Clinicopathological factors in relation to *AIB1* SNPs and diplotypes

Between 77% and 93% of the 576 patients were homozygous carriers of the common allele for each of the three *AIB1* SNPs. The four haplotypes were CGA (77.7%), CCA (11.3%), CGG (7.9%) and TGA (3.1%) (Fig. 1c). The CGA_CGA diplotype, i.e. homozygous common alleles in all three SNPs, accounted for 61.4% of all diplotypes, followed by CGA_CCA (16.4%), CGA_CGG (12.0%) and ‘Rare’ (10.2%) (Table 1). The genotype frequencies are in line with those reported in the 1000Genomes European population, with a maximum difference of 0.5%.

We first investigated whether *AIB1* genotypes were significantly associated with clinicopathological features (Supplementary Tables 1, 2 for all 576 patients and Supplementary Tables 3, 4 for 503 patients with ER^+^ tumors). Patients with the AIB1_2 any C allele were more often ≥ 50 years compared to G/G carriers and also more likely to have ER^+^ tumors. Patients with the AIB1_3 any G allele were more likely to be overweight/obese compared to A/A carriers. The common diplotype CGA_CGA was associated with increased frequency of triple negative breast cancer compared with other diplotypes. Patients with CGA_CCA were more often ≥ 50 years and more likely to have ER^+^ or PR^+^ tumors. Diplotype CGA_CGG carriers were more likely to be overweight/obese compared to patients with other diplotypes. All *P*_s_ < 0.05.

### *AIB1* SNPs and prognosis in different treatment groups

Patients were followed for up to 15 years. The median follow-up for the 372 patients still at risk was 11.1 years (interquartile range 11.0–13.1 years). During the last follow-up by June 30, 2019, a total of 144 of the 576 patients had had a breast cancer event (46 locoregional, 32 contralateral, 93 distant metastasis), of which 77 subsequently died. An additional 60 patients died without a previous breast cancer event.

**Table 1:** Patient and tumor characteristics at inclusion, treatments, and breast cancer events

	All patients		Patients with ER^+^ tumors	
	n = 576		n = 503	
	n (%)		n (%)	
Patient characteristics		Missing		Missing
Age ≥ 50 years	456 (79.2)		406 (80.7)	
BMI ≥ 25 kg/m^2^	270 (41.7)	3	227 (45.4)	3
Nulliparous	84 (14.6)		76 (15.1)	
Current smoker	121 (21.0)		102 (20.3)	
Coffee intake ≥ 2 cups/day	477 (82.8)		411 (81.7)	
Alcohol abstainer	63 (10.9)		50 (9.9)	
Ever use of MHT	267 (46.4)	1	244 (48.6)	1
*AIB1* genotypes				
rs6094752		2		2
CC	542 (94.4)		471 (94.0)	
CT	29 (5.1)		27 (5.4)	
TT	3 (0.5)		3 (0.6)	
rs2230782		7		7
GG	453 (79.6)		387 (78.0)	
GC	104 (18.3)		97 (19.6)	
CC	12 (2.1)		12 (2.4)	
rs2076546		2		2
AA	489 (85.2)		431 (86.0)	
AG	79 (13.8)		66 (13.2)	
GG	6 (1.0)		4 (0.8)	
Diplotypes		9		9
CGA_CGA	348 (61.4)		296 (59.9)	
CGA_CCA	93 (16.4)		88 (17.8)	
CGA_CGG	68 (12.0)		58 (11.7)	
Rare	58 (10.2)		52 (10.7)	
Tumor characteristics				
Invasive tumor size				
1–20 mm	423 (73.4)		376 (74.8)	
> 20 mm or skin/muscle involvement	153 (26.6)		127 (25.2)	
Any axillary node involvement	221 (38.5)	2	189 (37.7)	2
Histological grade		1		
I-II	462 (80.3)		440 (87.5)	
III	113 (19.7)		63 (12.5)	
Hormone receptor status				
ER^+^	503 (87.5)	1	503 (100)	
PR^+^	401 (69.7)	1	397 (78.9)	
HER2^+^	59 (11.5)	62	34 (7.5)	51
Triple negative	38 (6.7)	7	0 (0)	
Final surgical technique				
Mastectomy	236 (41.0)		206 (41.0)	
Adjuvant treatments				
Radiotherapy	350 (60.8)		297 (59.0)	
Chemotherapy	106 (18.4)		58 (11.5)	
Endocrine therapy (ER^+^ only, n = 503)				
Ever tamoxifen	313 (62.2)		313 (62.2)	
Ever aromatase inhibitor	206 (41.0)		206 (41.0)	
Type of event				
Any breast cancer event	144 (25)		123 (24.5)	
Any locoregional	46 (8)		42 (8.3)	
Any contralateral	32 (5.6)		27 (5.4)	
Any distant metastasis	93 (16.1)		78 (15.5)	
Death due to any cause	137 (23.8)		112 (22.3)	

BMI: Body mass index; MHT: Menopausal hormone therapy; ER^+^: Estrogen receptor positive; PR^+^: Progesterone receptor positive; HER2^+^: Human epidermal growth factor receptor 2 amplified

There were no significant associations with prognosis for the individual SNPs in the univariable or multivariable analyses. Adjustments for final type of surgery yielded essentially the same results. Interaction analyses were carried out to evaluate if the SNPs carried differential prognostic information depending on radiotherapy or chemotherapy. There were no interactions between any of the three SNPs and chemotherapy in regards to any breast cancer event. An interaction was seen between the AIB1_1 any T and radiotherapy, which showed that the combination was associated with lower risk for any breast cancer event (*P*_interaction_ = 0.068; HR_adj_ 0.26). After stratification according to radiotherapy, SNP AIB1_1 any T was significantly associated with increased breast cancer events in patients not treated with radiotherapy (Log-Rank *P* = 0.017; HR_adj_ 3.44; 95% CI 1.47–8.04). This result held after adjustment for FDR. There was no difference according to AIB1_1 genotype in radiotherapy-treated patients either in univariable or multivariable models. There were no interactions between the remaining two *AIB1* SNPs and radiotherapy with respect to any breast cancer event.

To evaluate if the SNPs carried differential prognostic information depending on tamoxifen or AI, interaction analyses were carried out in 503 patients with ER^+^ tumors. The interaction analysis showed an interaction between AIB1_2 any C and tamoxifen treatment (*P*_interaction_ = 0.082; HR_adj_ 0.44). However, after stratification for tamoxifen, the AIB1_2 any C showed no associations with breast cancer events in either group. No significant interactions were observed between the three *AIB1* SNPs and AIs. Further adjustment for final type of surgery yielded essentially the same results.

### Interplay between *AIB1* diplotypes, chemotherapy and radiotherapy on prognosis

**Fig. 2:**
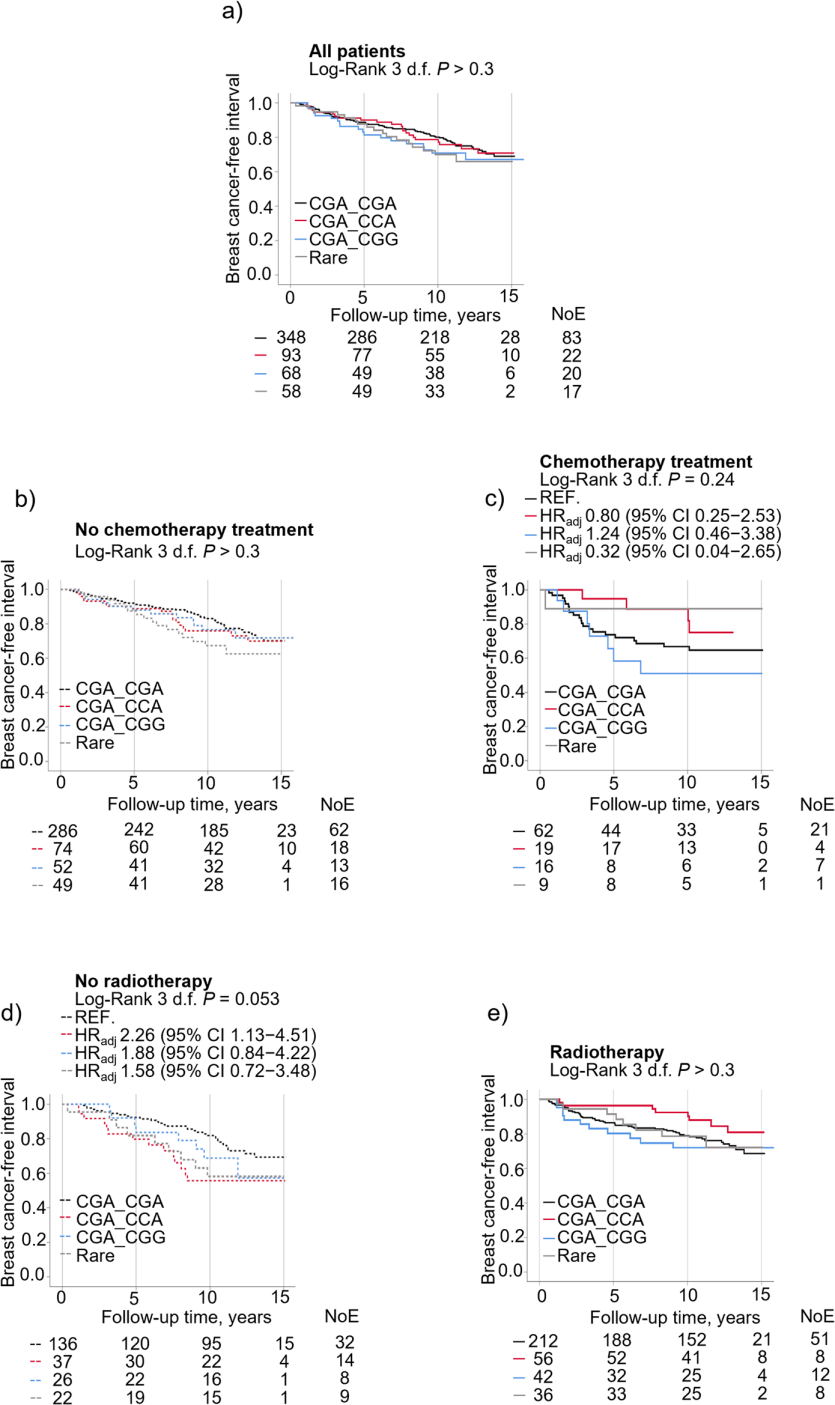
Kaplan-Meier curves showing breast cancer-free interval in patients according to diplotype (CGA_CGA, CGA_CCA, CGA_CGG and ‘Rare’) and treatments in (**a**) all patients. Breast cancer-free interval according to diplotype and chemotherapy status in (**b**) chemonaïve patients (**c**) chemotherapy-treated patients. Breast cancer-free interval according to diplotype and radiotherapy status in (**d**) non-radiotherapy-treated patients (**e**) radiotherapy-treated patients. Adjusted hazard ratios (HR_adj_) with 95% confidence intervals (CI) are presented for each group. Multivariable Cox regression models were adjusted for age at inclusion, BMI, tumor size, axillary lymph node involvement, histological grade, ER^+^, HER2^+^, and use of adjuvant treatments (chemotherapy, radiotherapy, tamoxifen and/or aromatase inhibitors). The number of patients at each follow-up and number of events (NoE) are indicated.

No significant association was observed between the different *AIB1* diplotypes and prognosis in all 576 patients (Fig. 2a). To investigate if *AIB1* diplotypes carried differential prognostic information depending on chemotherapy or radiotherapy, interaction analyses were carried out. There were no significant interactions between diplotypes and chemotherapy (Fig. 2b, c). There were interactions between radiotherapy and the diplotype CGA_CGA (*P*_interaction_ = 0.033) or CGA_CCA (*P*_interaction_ = 0.017) in relation to breast cancer events. In non-radiotherapy-treated patients, CGA_CGA carriers had the best prognosis, and CGA_CCA carriers the worst prognosis, with two-fold risk of breast cancer events in CGA_CCA compared with CGA_CGA carriers (Fig. 2d). In contrast, in radiotherapy-treated patients, CGA_CCA carriers had the best prognosis of all diplotype groups, although the differences were not statistically significant (Fig. 2e).

**Fig. 3:**
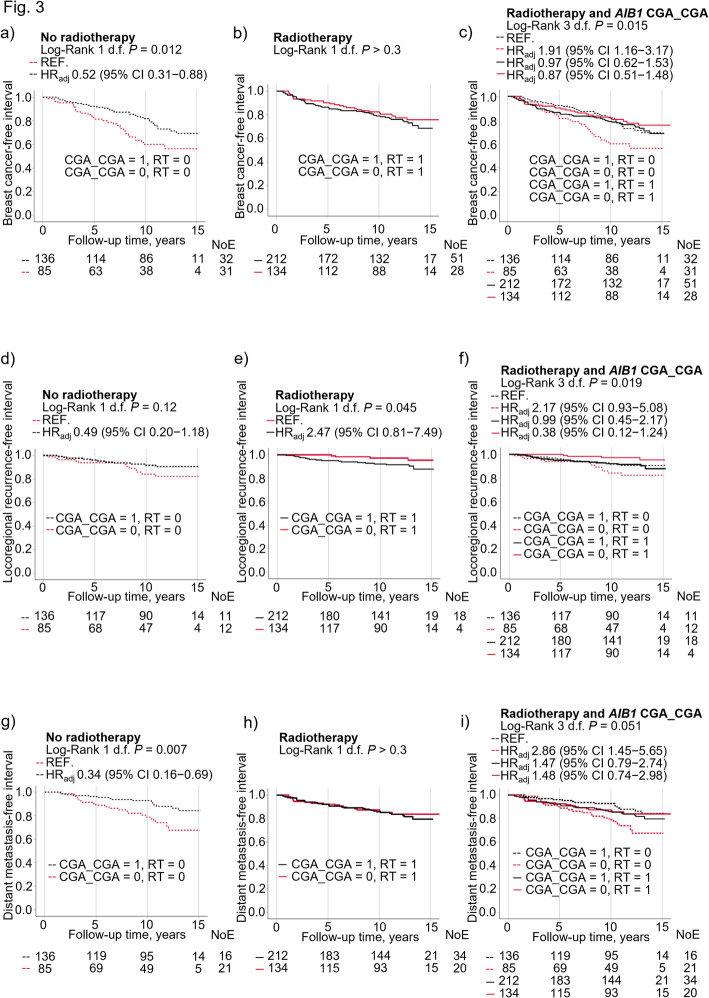
Kaplan-Meier curves showing that breast cancer-free interval differed according to CGA_CGA status and radiotherapy in (**a**) non-radiotherapy-treated (RT = 0) patients (**b**) radiotherapy-treated (RT = 1) patients (**c**) in four separate groups based on CGA_CGA and radiotherapy status. Locoregional recurrence-free interval did not differ in patients according to CGA_CGA and radiotherapy status (**d**) non-radiotherapy-treated patients (**e**) radiotherapy-treated patients (**f**) in four separate groups based on CGA_CGA and radiotherapy status. Distant metastasis-free interval differed in patients according to CGA_CGA and radiotherapy status in (**g**) non-radiotherapy-treated patients (**h**) radiotherapy-treated patients (**i**) four separate groups based on CGA_CGA and radiotherapy status. Adjusted hazard ratios (HR_adj_) with 95% confidence intervals (CI) are presented for each group. Multivariable Cox regression models were adjusted for age at inclusion, BMI, tumor size, axillary lymph node involvement, histological grade, ER^+^, HER2^+^, and use of adjuvant treatments (chemotherapy, radiotherapy, tamoxifen and/or aromatase inhibitors). The number of patients at each follow-up and number of events (NoE) are indicated.

Since there were significant interactions between radiotherapy and the two diplotypes CGA_CGA and CGA_CCA, these diplotypes were further investigated. Significant interactions were found between radiotherapy and the common diplotype CGA_CGA on any breast cancer event (*P*_interaction_ = 0.033; Fig. 3a, b, c; Full model in Supplementary Table 5), locoregional recurrence (*P*_interaction_ = 0.014; Fig. 3d, e, f), and distant metastasis (*P*_interaction_ = 0.022; Fig. 3g, h, i). The impact of the CGA_CGA diplotype in non-radiotherapy-treated patients was more pronounced with regards to distant metastasis than locoregional recurrence. When patients were divided into four groups based on CGA_CGA and radiotherapy status, the highest risk was seen in non-CGA_CGA carriers who had not received radiotherapy. Similar results were seen for locoregional recurrences. The differences became somewhat stronger after further adjustment for final type of surgery.

**Fig. 4:**
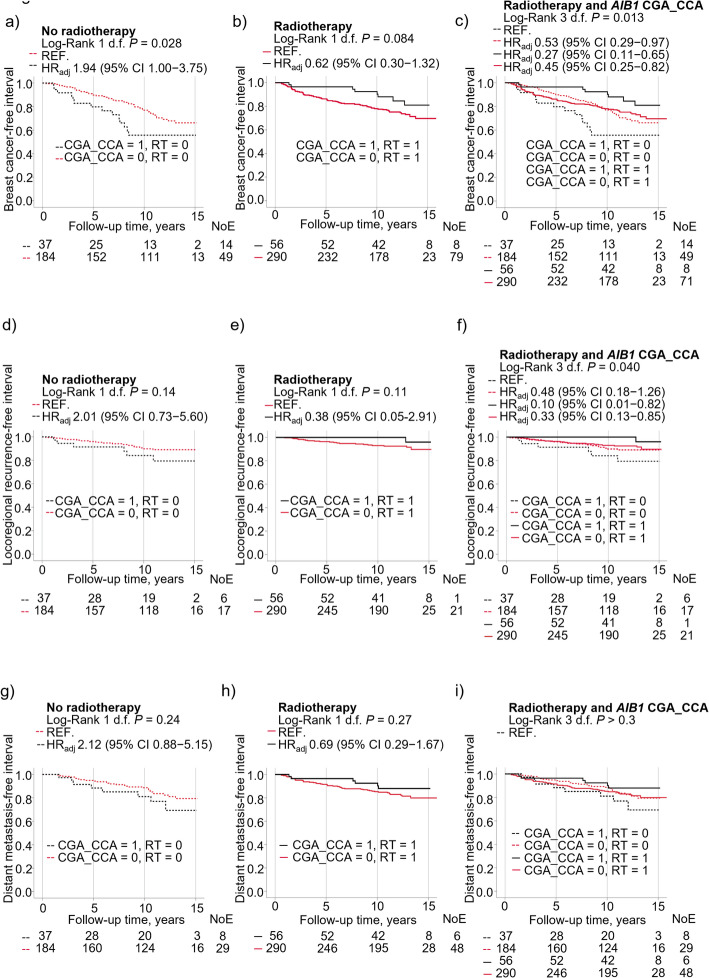
Kaplan-Meier curves showing that breast cancer-free interval in patients differed according to CGA_CCA status and radiotherapy in (**a**) non-radiotherapy-treated (RT = 0) patients (**b**) radiotherapy-treated (RT = 1) patients (**c**) in four separate groups based on CGA_CCA and radiotherapy status. Locoregional recurrence-free interval differed in patients according to CGA_CCA and radiotherapy status in (**d**) non-radiotherapy-treated patients (**e**) radiotherapy-treated patients (**f**) in four separate groups based on CGA_CCA and radiotherapy status. Distant metastasis-free interval in patients did not differ according to CGA_CCA and radiotherapy status in (**g**) non-radiotherapy-treated patients (**h**) radiotherapy-treated patients (**i**) in four separate groups based on CGA_CCA and radiotherapy status. Adjusted hazard ratios (HR_adj_) with 95% confidence intervals (CI) are presented for each group. Multivariable Cox regression models were adjusted for age at inclusion, BMI, tumor size, axillary lymph node involvement, histological grade, ER^+^, HER2^+^, and use of adjuvant treatments (chemotherapy, radiotherapy, tamoxifen and/or aromatase inhibitors). Number of patients at each follow-up and number of events (NoE) are indicated

The differences observed appeared to be driven by the CGA_CCA diplotype in non-CGA_CGA carriers. Interactions were found between the diplotype CGA_CCA and radiotherapy on breast cancer events for any event (*P*_interaction_ = 0.017; Fig. 4a, b, c; Supplementary Table 6), locoregional recurrence (*P*_interaction_ = 0.089; Fig. 4d, e, f) and distant metastasis (*P*_interaction_ = 0.088; Fig. 4g, h, i). Patients with CGA_CCA had poorer outcome if they were not treated with radiotherapy. However, in contrast to the analyses with CGA_CGA, the interactions for the CGA_CCA diplotype were strongest for any breast cancer event, while for CGA_CGA the interactions were strongest for locoregional recurrence. Therefore, the next step was to investigate other types of systemic therapy beyond chemotherapy including both types of endocrine therapy.

### The interplay between *AIB1* diplotypes, aromatase inhibitors and tamoxifen on prognosis in 503 patients with ER^+^ tumors

**Fig. 5:**
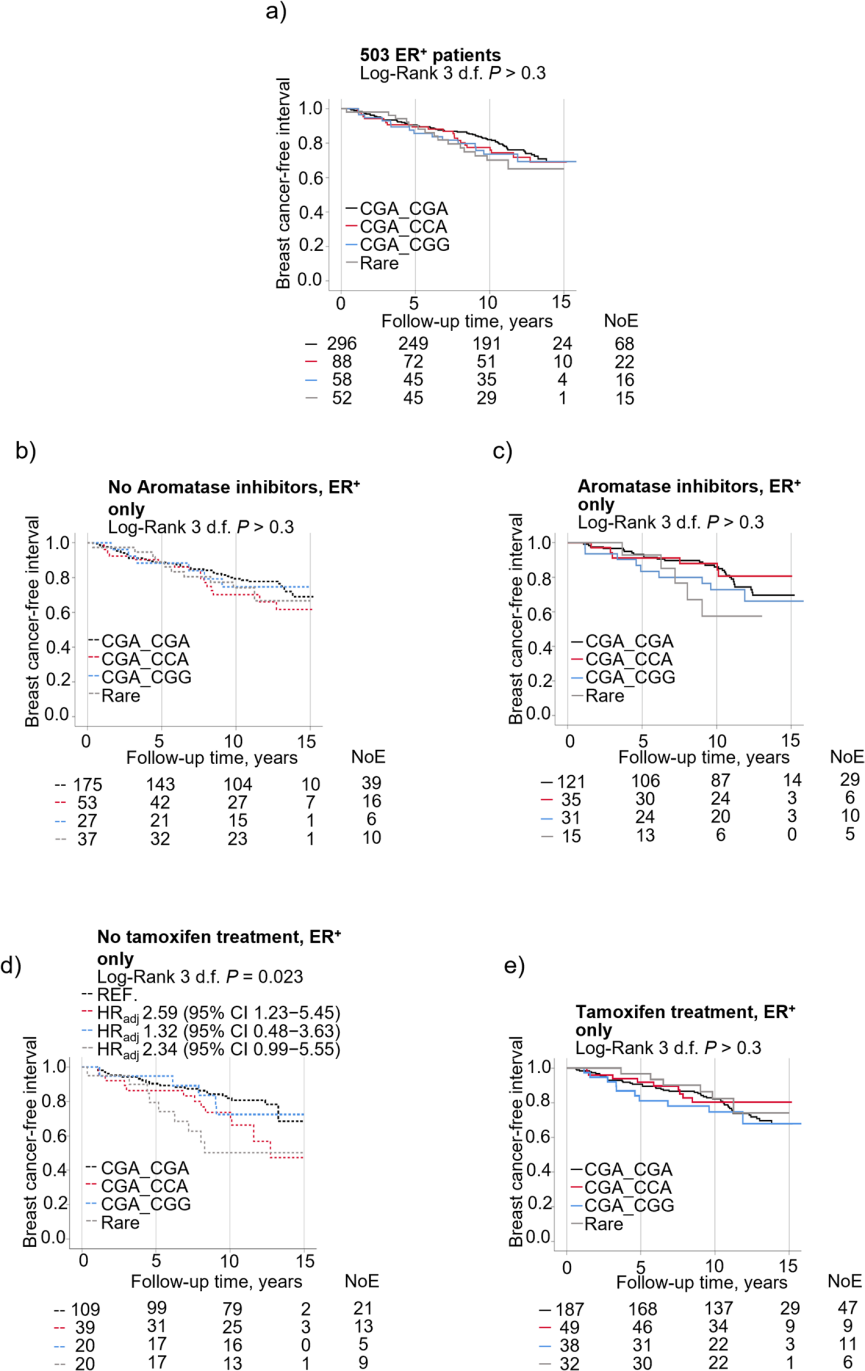
Kaplan-Meier curves showing that breast cancer-free interval did not differ in ER^**+**^ patients according to diplotype (CGA_CGA, CGA_CCA, CGA_CGG and ‘Rare’) in (**a**) all patients. Breast cancer-free interval according to diplotype and aromatase inhibitor status in (**b**) non-aromatase inhibitor treated patients (**c**) aromatase inhibitor-treated patients. Breast cancer-free interval according to diplotype and tamoxifen status in (**d**) non-tamoxifen-treated patients (**e**) tamoxifen-treated patients. Adjusted hazard ratios (HR_adj_) with 95% confidence intervals (CI) are presented for each group. Multivariable cox regression models were adjusted for age at inclusion, BMI, tumor size, axillary lymph node involvement, histological grade, ER^+^, HER2^+^, and use of adjuvant treatments (chemotherapy, radiotherapy, tamoxifen and/or aromatase inhibitors). Number of patients at each follow-up and number of events (NoE) are indicated.

Overall, there was no association between the different diplotypes and prognosis in the 503 patients with ER^+^ tumors (Fig. 5a). Interaction analyses were carried out to investigate if any diplotype carried differential prognostic information depending on type of endocrine treatment. No interaction was seen between AI treatment and any of the diplotypes on breast cancer events (Fig. 5b, c). There was an interaction between CGA_CGA and tamoxifen treatment on the risk of breast cancer events (*P*_interaction_ = 0.079; Supplementary Table 7). No interactions were observed for any of the diplotypes and tamoxifen. However, patients carrying CGA_CCA or ‘Rare’ diplotypes had a more than two-fold risk for breast cancer events in non-tamoxifen-treated patients compared to CGA_CGA (Fig. 5d). No significant associations were found between the diplotypes in tamoxifen-treated patients (Fig. 5e).

In patients not treated with tamoxifen, CGA_CGA-carriers had less than half the risk of any breast cancer event compared to non-CGA_CGA-carriers (Supplementary Fig. 1 A). No significant association was seen between CGA_CGA carriers and non-carriers and risk for breast cancer events in tamoxifen-treated patients (Supplementary Fig. 1B). When divided into four groups based on CGA_CGA and tamoxifen status, the worst outcome was seen in non-tamoxifen-treated patients with non-CGA_CGA diplotypes (Supplementary Fig. 1 C).

As for contralateral breast cancer, there was an interaction between CGA_CGA and tamoxifen (*P*_interaction_ = 0.055), with similar associations between CGA_CGA and tamoxifen status as for any breast cancer event (Supplementary Fig. 1D, E). When divided into four groups based on CGA_CGA and tamoxifen status, there was a significant difference between the groups in the univariable model, but not in the multivariable model (Supplementary Fig. 1 F). Regarding risk of distant metastasis there were no significant differences between CGA_CGA carriers and non-CGA_CGA-carries irrespective of tamoxifen status (Supplementary Fig. 1G, H, I).

Taken together, *AIB1* genotypes were associated with prognosis only when treatment was considered. There were nominally significant interactions between *AIB1* diplotypes and radiotherapy that appeared to be driven by AIB1_2. However, the interactions did not remain statistically significant after FDR adjustment. 

### Differential signaling pathways in *AIB1* mRNA high vs. low breast cancer tumors

Provided that others have shown that the three investigated functional SNPs are linked to mRNA expression, AIB1_1 and AIB1_2 to lower expression and AIB1_3 to higher expression, we used data from the SCAN-B cohort to elucidate potential biological processes and pathways associated with *AIB1* mRNA expression.

In the SCAN-B cohort, biological processes that differed between *AIB1* high (tertile 3 (T3)) vs. low (tertile 1 (T1)) mRNA expressing tumors were examined. These samples contained both tumor cells and cells from the tumor microenvironment (TME). The up- and downregulation of associated genes was assessed separately by tumor ER status (Supplementary Fig. 2 A, B). In ER^+^ tumors a total of 96 genes were upregulated in high *AIB1* expressing vs. low *AIB1* expressing tumors while a total of 34 genes were downregulated (Supplementary Table 8). In ER^-^ tumors, a total of 322 genes were upregulated in high *AIB1* expressing vs. low *AIB1* expressing tumors and 145 genes were downregulated (Supplementary Table 9). In both ER categories, gene sets related to Interferon alpha and gamma response, allograft rejection, PI3K/AKT/MTOR signaling, IL6/JAK/STAT3 signaling, inflammatory response, complement and IL2/STAT5 signaling were activated in high vs. low *AIB1* mRNA tumors. In both ER categories, genes related to EMT and myogenesis were suppressed in *AIB1* mRNA high vs. low tumors indicating lower risk for distant metastasis. High *AIB1* was associated with activated E2F, MYC targets and G2M checkpoint in ER^+^ breast cancers but suppressed in ER^-^ breast cancers. The estrogen response was suppressed in *AIB1* mRNA high expression tumors, only in ER^+^ tumors (Supplementary Fig. 2 C, D, Supplementary Tables 10,11). This shows that the impact of *AIB1* mRNA expression differs between ER^+^ and ER^-^ breast cancers.

Integrating the results from the GSEA and hallmark signature annotations analyses with the genotype data, our findings suggest that low *AIB1* mRNA expression (linked to the AIB1_2 any C that is part of the CGA_CCA diplotype) confers low IL-6 signaling leading to higher radiosensitivity. At the same time, low AIB1 mRNA expression may confer more EMT. This might explain the interaction between radiotherapy and CGA_CCA as schematically illustrated in (Supplementary Fig. 3).

## Discussion

Our main finding was that there were nominally significant interactions between *AIB1* genotypes and radiotherapy on risk for breast cancer events. *AIB1* genotypes were associated with prognosis only when treatment was considered. To our knowledge there are no previous studies investigating the impact of *AIB1* genotypes on radiotherapy or tamoxifen response in breast cancer patients.

In this study, radiotherapy was given as adjuvant treatment after surgery. Therefore, normal cells, residual tumor cells, and remaining cells from the TME were irradiated, rather than the primary tumor. Studies have shown how different cells within the TME can affect cancer cells’ response to radiotherapy [41, 42]. One study demonstrated how cancer associated adipocytes promote breast cancer radioresistance [43]. Another study found that cancer-associated fibroblasts drive growth and confer radioresistance in breast cancer cells through paracrine signaling of IL-6 [44]. *AIB1* can promote the production of IL-6 through nuclear factor-κB (NF-κB) [45–47]. Results from the SCAN-B cohort showed that genes related to IL-6 were upregulated in *AIB1* high tumors compared with *AIB1* low tumors. AIB1_2 any C, which is part of CGA_CCA, has been associated with lower *AIB1* expression [18] and possibly lower IL-6. Lower IL-6 levels could decrease radioresistance in the remaining breast cancer cells and could be one explanation for the lower rates of breast cancer events after radiotherapy in CGA_CCA carriers. Moreover, the SCAN-B results generally showed that high *AIB1* mRNA was associated with increased immune system activation and greater EMT suppression compared with lower *AIB1* expression. This is in contrast to earlier research, which showed that high AIB1 contributed to a pro-EMT phenotype [9]. However, if indeed low levels, which are linked to CGA_CCA, have decreased immune activation and higher EMT, this could explain why CGA_CCA carriers who had not received radiotherapy had a worse prognosis.

The variant allele of AIB1_3 any G has been shown to increase the amount of *AIB1* mRNA in several different tissues i.e. whole blood [18]. Based on our hypothesis, it could be assumed that CGA_CGG confers higher levels of *AIB1* mRNA than CGA_CGA and therefore also an increased resistance to radiotherapy. Considering the small portion of patients carrying the CGA_CGG diplotype, a larger cohort might help clarify if CGA_CGG confers radiotherapy resistance.

The only SNP where an interaction was seen was AIB1_1 any T. This SNP was associated with greater risk of events in patients not treated with radiotherapy and has, like AIB1_2, been associated with lower *AIB1* mRNA. This could contribute to the results seen with non-CGA_CGA diplotypes, but since all patients with any T were included in the ‘Rare’ group, they were not examined separately. Larger sample sizes would be needed to analyze diplotypes carrying AIB1_1 any T.

It is possible that the observed interactions between *AIB1* genotypes and radiotherapy are due to linkage disequilibrium, where the functional SNPs responsible for the associations are in tight linkage with the studied *AIB1* SNPs [48]. Using LDexpress, all three SNPs were found to be in linkage disequilibrium with numerous other SNPs within a ± 50,000 bp window both within the *AIB1* gene and adjacent regions [18]. Genetic variation in other genes has previously been associated with radiotherapy response including side-effects but *AIB1* was not included [30, 49]. Further, genomic profiling of the tumor may also aid in determining who would benefit from radiotherapy and who can safely forego radiotherapy even after breast conserving surgery [50, 51]. Today, the cost of targeted genotyping from an easily obtained blood sample is low compared to genomic profiling of the tumor and can be done well in advance of selecting type of surgery as well as adjuvant treatments. There are still no clinically used or validated pharmacogenomic predictors for radiotherapy response.

Breast cancer treatments changed since the study started in 2002. There has been an increasing proportion of patients receiving breast conserving surgery followed by radiotherapy since 2002, when approximately 60% of patients received breast conserving surgery. Recent studies have found breast conserving surgery followed by radiotherapy to confer better prognosis than mastectomy irrespective of radiotherapy [52, 53]. In 2023, about 75% of patients were operated on with breast conserving techniques, of which about 95% received postoperative radiotherapy, according to the Swedish National quality registry for breast cancer. We believe that the increased utilization of radiotherapy in breast cancer treatment could increase the importance of our findings if confirmed.

This study has some limitations. A lack of research nurses at the time of patients’ preoperative visits was the main reason behind the non-included patients. Approximately 5% of operated patients had an unverified diagnosis at the time of surgery and were therefore not included in the study. Some patients were excluded due to poor Swedish understanding. Although there was no explicit question on ethnicity, most included patients were of European ancestry and the results need validation in patients with other backgrounds. The genotype frequencies aligned with those reported in the 1000Genomes European population. Although there were many nominally significant *P*-values, the interactions did not hold after FDR adjustment. After stratification, some subgroups were small. However, this is an exploratory study where nominal *P*-values can be used. Positive results in explorative studies could be due to chance, should be interpreted with caution and need to be confirmed in further targeted studies [54].

Another limitation of this study is that the SCAN-B–based pathway findings could not be externally replicated, as no comparable real-world RNA-seq dataset with matching processing and clinical annotation exists. Although SCAN-B includes rigorous normalization and protocol-adjustment steps, the absence of an independent cohort means these results should be interpreted with caution. As the analyses were exploratory and aimed at improving biological insight, the identified pathways should be viewed as hypothesis-generating and as a basis for future mechanistic studies.

This study has several strengths. Firstly, the use of a population-based prospective cohort with long follow-up and near complete data on clinicopathological and patient characteristics. Since the first questionnaires were completed prior to surgery there was minimal risk of recall bias regarding clinical outcome. Approximately 10% of the genotypes tested with iPlex were tested in duplicate on different plates. The duplicates were in 100% agreement with the original samples, which implies high reliability of the method. If there is a causal link between *AIB1* genotypes and the prognostic impact depending on the type of adjuvant treatment, the results should be consistent in other patient populations.

Since the adjuvant treatments were based on tumor characteristics and not randomized, the genotypes were investigated within different treatment groups rather than looking at what treatment yielded the best prognosis for each genotype. It would be interesting to study these genotypes in an existing randomized trial of adjuvant radiotherapy.

## Conclusions

In conclusion, host *AIB1* genotypes conferred differential prognostic impact depending on adjuvant radiotherapy status. Patients with the CGA_CCA diplotype appeared to derive the highest benefit from radiotherapy. If confirmed in independent datasets, *AIB1* merits further evaluation as a putative pharmacogenetic marker to identify breast cancer patients that benefit the most from radiotherapy. The cost of genotyping is currently very low compared to the cost of ineffective or harmful cancer treatment. Taking host factors, including genetic variants, into account may improve treatment selection.

## Supplementary Information

Supplementary Material 1.

Supplementary Material 2.

## Data Availability

Clinical data are not publicly available due to privacy laws. Questions regarding data can be directed to the corresponding author.

## Abbreviations

AI: Aromatase inhibitor
AIB1: Amplified in breast cancer 1
BMI: Body mass index
CI: Confidence interval
DEGs: Differentially expressed genes
DGE: Differential gene expression
DNA: Deoxyribonucleic acid
EMT: Epithelial mesenchymal transition
ER: Estrogen receptor
FDR: False discovery rate
GSEA: Gene set enrichment analysis
HER2: Human epidermal growth factor 2
HR: Hazard ratio
IGF1: Insulin like growth factor–1
Log_2_ FC: Log_2_ fold change
NF–κB: Nuclear factor–κB
OR: Odds ratio
PR: Progesterone receptor
SNP: Single nucleotide polymorphism
TME: Tumor microenvironment
