# Supplementary material for: Interplay between AIB1 genotypes and radiotherapy in a Swedish population-based breast cancer cohort
Source: Discov Oncol. 2026 Jan 3;17:159. doi: 10.1007/s12672-025-04370-6 (PMC12847612; doi:10.1007/s12672-025-04370-6)
Supplement: Supplementary file 1 — Supplementary Material 1. [file 12672_2025_4370_MOESM1_ESM.pdf]

Interplay between *AIB1* genotypes and radiotherapy in a Swedish population-based breast cancer cohort

Alexandra Wiberg*[,1], Louise Ebbesen*[,1], Christopher Godina [1], Karolin Isaksson [2,3], Helena Jernström [1,✉]

1 Division of Oncology, Department of Clinical Sciences Lund, Lund University Cancer Center/Kamprad, Lund University and Skåne University Hospital, Barngatan 4, SE-221 85 Lund, Sweden

2 Division of Surgery, Department of Clinical Sciences Lund, Lund University Cancer Center, Lund University, SE-221 85 Lund, Sweden

3 Department of Surgery, Skåne University Hospital, Kristianstad, J A Hedlunds väg 5, SE-291 33 Kristianstad, Sweden*Shared first authors

Corresponding author: Helena Jernström, helena.jernstrom@med.lu.se

Table of contents

Supplementary Figure 1 2

Supplementary Figure 2 3

Supplementary Figure 3 4

Supplementary Table 1 5

Supplementary Table 2 6

Supplementary Table 3 7

Supplementary Table 4 8

Supplementary Table 5 9

Supplementary Table 6 10

Supplementary Table 7 11

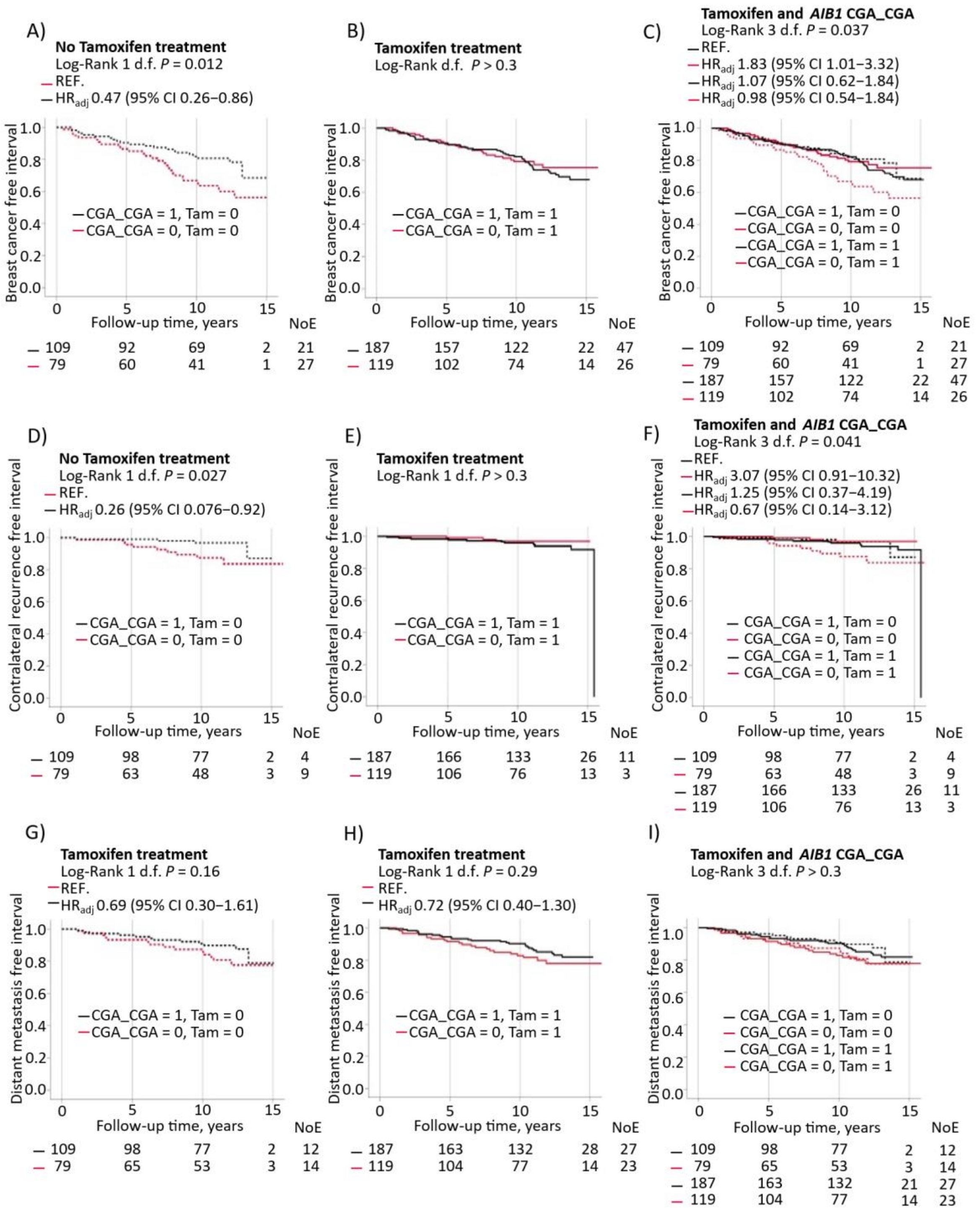

Supplementary Figure 1. Kaplan-Meier curves showing breast cancer free interval in patients according to CGA_CGA status in **A)** non-tamoxifen-treated (Tam=0) patients **B)** tamoxifen-treated (Tam=1) patients **C)** in four separate groups based on tamoxifen and CGA_CGA. Contralateral recurrence free interval in patients according to CGA_CGA status in **D)** non-tamoxifen-treated patients **E)** tamoxifen-treated patients **F)** in four separate groups based on CGA_CGA and tamoxifen status. Distant metastasis free interval in patients according to CGA_CGA status in **G)** non-tamoxifen-treated patients **H)** tamoxifen-treated patients **I)** in four separate groups based on tamoxifen and CGA_CGA. Adjusted hazard ratios (HR_{adj}) with 95% confidence interval (CI) are presented for each group. Multivariable cox regression models were adjusted for age at inclusion, BMI, tumor size, axillary lymph node involvement, histological grade, ER^+, $HER2^+$, and use of adjuvant treatments (chemotherapy, radiotherapy, tamoxifen and/or aromatase inhibitors). Number of patients at each follow-up and number of events (NoE) are indicated.

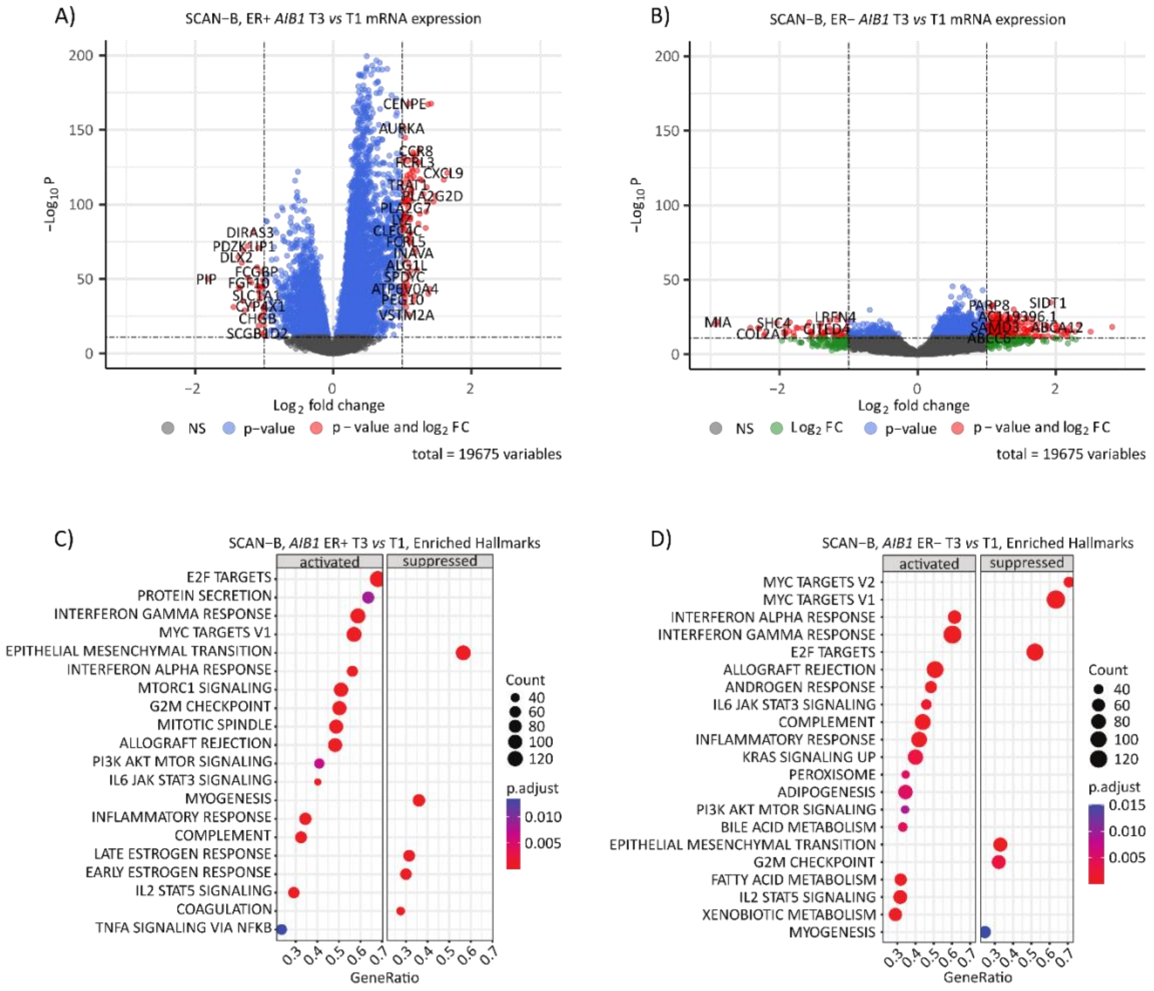

Supplementary Figure 2. Volcano plots showing up- and downregulated genes in tumors from the highest (T3) *vs* lowest (T1) *AIB1* mRNA tertiles in tumors from SCAN-B, by false discovery rate (FDR)-adjusted *P*-values and fold change (Log_2 FC). Genes marked in red are counted as differentially expressed with a FDR ≥0.05 and Log_2 FC ≥1.0 for up-regulated genes or ≤-1 for down-regulated genes. Blue genes only fulfill the FDR criteria, green genes only fulfill the Log_2 FC criteria, and gray genes fulfill neither A) in estrogen receptor positive (ER^+) tumors B) in estrogen receptor negative (ER^-) tumors. Dot plots showing activated and suppressed Hallmark Signatures in T3 vs T1 *AIB1* mRNA tertiles in C) ER^+ tumors D) ER^- tumors.

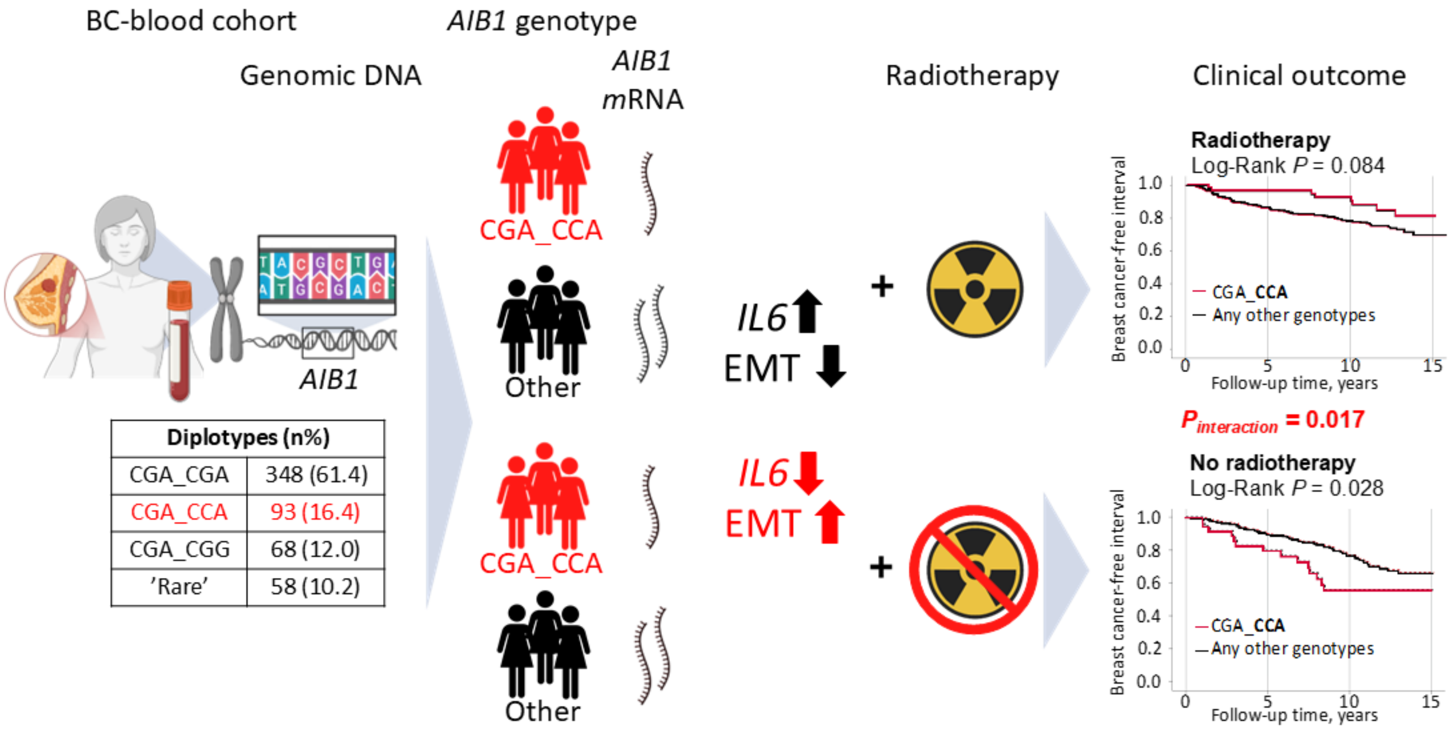

Supplementary Figure 3. Schematic figure illustrating the proposed mechanism for the interaction between the CGA_CCA diplotype and radiotherapy on breast cancer-free interval. **IL-6** – Interleukin 6, **EMT** – Epithelial mesenchymal transition

Partially created with BioRender.

Supplementary Table 1. Clinicopathological factors, treatments and breast cancer events in relation to *AIB1* SNPs in 576 breast cancer patients

	All patients		*AIB1* (C218R, 652C>T) rs6094752		*AIB1* Q586H (1758G>C) rs2230782		*AIB1* T960T (2880A>G) rs2076546	
			C/C	Any T	G/G	Any C	A/A	Any G
	n = 576	Missing	n = 542 (94.4)	n = 32 (5.6)	n = 453 (79.6)	n = 116 (20.4)	n = 489 (85.2)	n = 85 (14.8)
	n (%)		n (%)	n (%)	n (%)	n (%)	n (%)	n (%)
**Patient characteristics**								
Age ≥ 50 years	456 (79.2)		429 (79.2)	25 (78.1)	347 (76.6)	102 (87.9)	390 (79.8)	64 (75.3)
BMI ≥ 25 kg/m^2	270 (41.7)	3	255 (47.1)	13 (43.3)	207 (45.9)	56 (48.7)	218 (44.8)	50 (59.5)
Nulliparous	84 (14.6)		79 (14.6)	5 (15.6)	65 (14.3)	17 (14.7)	72 (14.7)	12 (14.1)
Current smoker	121 (21.0)		116 (21.4)	5 (15.6)	98 (21.6)	23 (19.8)	99 (20.2)	22 (25.9)
Coffee intake ≥ 2 cups/day	477 (82.8)		449 (82.8)	27 (84.4)	369 (81.5)	102 (87.9)	406 (83.0)	70 (82.4)
Alcohol abstainer	63 (10.9)		58 (10.7)	4 (12.5)	51 (11.3)	12 (10.3)	48 (9.8)	14 (16.5)
Ever use of MHT	265 (46.1)	1	252 (46.6)	14 (43.8)	205 (45.3)	57 (49.6)	226 (46.9)	40 (47.1)
**Tumor characteristics**								
Invasive tumor size								
>20 mm or skin and muscle involvement	153 (26.6)		146 (26.9)	6 (18.8)	123 (27.2)	29 (25.0)	125 (25.6)	27 (31.8)
Any axillary node involvement	221 (38.5)	2	210 (38.9)	11 (34.4)	174 (38.6)	43 (37.1)	184 (37.8)	37 (43.5)
Histological grade III	113 (19.7)	1	107 (19.8)	6 (18.8)	91 (20.1)	20 (17.2)	96 (19.6)	17 (20.2)
Hormone receptor status								
ER^+	503 (87.3)		471 (86.9)	30 (93.8)	387 (85.4)	109 (94.0)	431 (88.1)	70 (82.4)
PR^+	401 (69.6)		373 (68.8)	26 (81.3)	310 (68.4)	86 (74.1)	343 (70.1)	56 (65.9)
$HER2^+$	59 (11.5)	62	56 (11.6)	3 (10.3)	46 (11.5)	13 (12.0)	47 (10.7)	12(16.7)
Triple negative	39 (6.8)	6	38 (7.1)	1 (3.1)	36 (8.0)	3 (2.6)	33 (6.8)	6 (7.3)
**Final surgical technique**								
Mastectomy	236 (41.0)		221 (40.8)	14 (43.8)	191 (42.2)	43 (37.1)	202 (41.3)	33 (38.8)
**Adjuvant treatments**								
Radiotherapy	350 (60.8)		330 (60.9)	19 (59.4)	276 (60.9)	71 (61.2)	297 (60.7)	52 (61.2)
Chemotherapy	105 (18.2)		100 (18.5)	5 (15.6)	83 (18.3)	22 (19.0)	85 (17.4)	20 (23.5)
Endocrine therapy (ER^+ only n = 503)								
Ever tamoxifen	313 (62.2)		295 (62.6)	17 (56.7)	244 (63.0)	63 (57.8)	265 (61.5)	47 (67.1)
Ever aromatase inhibitor	206 (41.0)		198 (42.0)	8 (26.7)	159 (41.1)	43 (39.4)	172 (39.9)	34 (48.6)
**Type of event**								
Any breast cancer event	144 (25)		133 (24.5)	10 (31.3)	117 (25.8)	26 (22.4)	118 (24.1)	25 (29.4)
Any locoregional	46 (8)		44 (8.1)	2 (6.3)	38 (8.4)	7 (6.0)	39 (8.0)	7 (8.2)
Any contralateral	32 (5.6)		29 (5.4)	3 (9.4)	25 (5.5)	7 (6.0)	28 (5.7)	4 (4.7)
Any distant metastasis	93 (16.1)		84 (15.5)	8 (25)	75 (16.6)	17 (14.7)	74 (15.1)	18 (21.2)
Death due to any cause	137 (23.8)		124 (22.9)	12 (37.5)	112 (24.7)	23 (19.8)	112 (22.9)	24 (28.2)

Single nucleotide polymorphisms (SNPs), Body mass index (BMI), Menopausal hormone therapy (MHT), Estrogen receptor positive (ER^+), Progesterone receptor positive (PR^+), Human epidermal growth factor receptor 2 amplified ($HER2^+$)

Supplementary Table 2. Clinicopathological factors, treatments and breast cancer events in relation to AIB1 diplotypes in 576 breast cancer patients

	All patients	Missing	CGA_CGA		CGA_CCA		CGA_CGG		Rare		Missing
	n = 576 (%)		Yes n =296 (%)	No n=198 (%)	Yes n=88 (%)	No n=406 (%)	Yes n=58 (%)	No n=436 (%)	Yes n=52 (%)	No n=442 (%)	n = 9 (%)
**Patients characteristics**											
Age ≥ 50 years	456 (79.2)		268 (77.0)	179 (81.7)	82 (88.2)	365 (77.0)	50 (73.5)	397 (79.6)	47 (81.0)	400 (78.6)	9 (100.0)
BMI ≥ 25 kg/m^2	270 (41.7)	3	150 (43.1)	111 (51.4)	46 (49.5)	215 (45.6)	40 (59.7)	221 (44.5)	25 (44.6)	236 (46.5)	9 (100.0)
Nulliparous	84 (14.6)		51 (14.7)	31 (14.2)	12 (12.9)	70 (14.8)	9 (13.2)	73 (14.6)	10 (17.2)	72 (14.1)	2 (22.2)
Current smoker	121 (21.0)		73 (21.0)	48 (21.9)	19 (20.4)	102 (21.5)	19 (27.9)	102 (20.4)	10 (17.2)	111 (21.8)	0 (0)
Coffee intake ≥ 2 cups/day	477 (82.8)		284 (81.6)	186 (84.9)	81 (87.1)	389 (82.1)	54 (79.4)	416 (83.4)	51 (87.9)	419 (82.3)	7 (77.8)
Alcohol abstainer	63 (10.9)		35 (10.1)	27 (12.3)	10 (10.8)	52 (11.0)	11 (16.2)	51 (10.2)	6 (10.3)	56 (11.0)	
Ever use of MHT	265 (46.1)	1	157 (45.1)	102 (46.8)	41 (44.6)	218 (46.0)	30 (44.1)	229 (46.0)	31 (53.4)	228 (44.9)	6 (66.7)
**Tumor characteristics**											
Invasive tumor size											
>20 mm or skin and muscle involvement	153 (26.6)		57 (26.0)	23 (24.7)	128 (27.0)	21 (30.9)	130 (26.1)	13 (22.4)	138 (27.1)	113 (25.6)	2 (22.2)
Any axillary node involvement	221 (38.5)	2	86 (39.3)	35 (37.6)	182 (38.6)	33 (48.5)	184 (37.0)	18 (31.8)	199 (39.3)	1067 (38.0)	4 (55.6)
Histological grade III	113 (19.7)	1	36 (16.5)	12 (12.9)	99 (20.9)	10 (14.9)	101 (20.2)	14 (24.1)	97 (19.1)	52 (11.8)	2 (22.2)
Hormone receptor status											
ER^+	503 (87.3)		296 (85.1)	198 (90.4)	88 (94.6)	406 (85.7)	58 (85.3)	436 (87.4)	52 (89.7)	442 (86.8)	9 (100.0)
PR^+	401 (69.6)		235 (67.5)	159 (72.6)	73 (78.5)	321 (67.7)	46 (67.6)	348 (69.7)	40 (69.0)	354 (69.5)	7 (77.8)
$HER2^+$	59 (11.5)	62	34 (10.9)	25 (12.8)	9 (10.5)	50 (11.8)	9 (16.4)	50 (11.0)	7 (13.0)	52 (11.5)	0 (0.0)
Triple negative	39 (6.8)	6	30 (8.7)	9 (4.2)	2 (2.2)	37 (7.9)	3 (4.6)	36 (7.3)	4 (6.9)	35 (7.0)	0 (0.0)
**Final surgical technique**											
Mastectomy			148 (42.5)	85 (38.8)	35 (37.6)	198 (41.8)	29 (42.6)	204 (40.9)	21 (36.2)	212 (41.7)	0 (0.0)
**Adjuvant treatments**											
Radiotherapy	350 (60.8)		212 (60.9)	134 (61.2)	56 (60.2)	290 (61.2)	42 (61.8)	304 (60.9)	36 (62.1)	310 (60.9)	4 (44.4)
Chemotherapy	105 (18.2)		62 (17.8)	43 (19.6)	18 (19.4)	87 (18.4)	16 (23.5)	89 (17.8)	9 (15.5)	96 (18.9)	0 (0.0)
Endocrine therapy (ER^+ only, n = 503)											
Ever tamoxifen	313 (62.2)		187 (63.2)	119 (60.2)	49 (55.7)	257 (63.3)	38 (65.5)	268 (61.5)	32 (61.5)	274 (62.0)	7 (77.8)
Ever AI	206 (41.0)		121 (40.9)	81 (40.9)	35 (39.8)	167 (41.1)	31 (53.4)	171 (39.2)	15 (28.8)	187 (42.3)	4 (44.4)
**Type of event**											
Any breast cancer event	144 (25)		83 (23.9)	265 (76.1)	22 (23.7)	71 (76.3)	20 (29.4)	48 (70.6)	17 (29.3)	41 (70.7)	2 (22.2)
Any locoregional	46 (8)		29 (8.3)	319 (91.7)	7 (7.5)	86 (92.5)	6 (8.8)	62 (91.2)	3 (5.2)	55 (94.8)	1 (11.1)
Any contralateral	32 (5.6)		18 (5.2)	330 (94.8)	6 (6.5)	87 (93.5)	2 (2.9)	66 (97.1)	6 (10.3)	52 (89.7)	0 (0.0)
Any distant metastasis	93 (16.1)		50 (14.4)	298 (85.6)	14 (15.1)	79 (84.9)	16 (23.5)	52 (76.5)	11 (19.0)	47 (81.0)	2 (22.2)
Death due to any cause	137 (23.8)		81 (23.3)	267 (76.7)	18 (19.4)	75 (80.6)	20 (29.4)	48 (70.6)	15 (25.9)	43 (74.1)	3 (33.3)

Body mass index (BMI), Menopausal hormone therapy (MHT), Estrogen receptor positive (ER^+), Progesterone receptor positive (PR^+), Human epidermal growth factor receptor 2 amplified ($HER2^+$)

Supplementary Table 3. Clinicopathological factors, treatments and breast cancer events in relation to *AIB1* SNPs in 503 patients with ER^+ tumors

	**Patients with ER^+ tumors** n = 503 n (%)	Missing	***AIB1* (C218R, 652C>T) rs6094752** C/C n = 471 n (%)	Any T n = 30 n (%)	***AIB1* Q586H (1758G>C) rs2230782** G/G n = 387 n (%)	Any C n = 109 n (%)	***AIB1* T960T (2880A>G) rs2076546** A/A n = 431 n (%)	Any G n = 70 n (%)
**Patient characteristics**								
Age ≥ 50 years	406 (80.7)		379 (80.5)	25 (83.3)	303 (78.3)	96 (88.1)	348 (80.7)	56 (80.8)
BMI ≥ 25 kg/m^2	227 (45.4)	3	214 (45.5)	11 (39.3)	167 (43.4)	53 (49.1)	187 (43.6)	38 (55.1)
Nulliparous	76 (15.1)		71 (15.1)	5 (16.7)	57 (14.7)	17 (15.6)	65 (15.1)	11 (15.7)
Current smoker	102 (20.3)		97 (20.6)	5 (16.7)	82 (21.2)	20 (18.3)	87 (20.2)	15 (21.4)
Coffee intake ≥ 2 cups/day	411 (81.7)		385 (81.7)	25 (83.3)	310 (80.1)	95 (87.2)	355 (82.4)	55 (78.6)
Alcohol abstainer	50 (9.9)		46 (9.8)	3 (10.0)	39 (10.1)	11 (10.1)	40 (9.3)	9 (12.9)
Ever use of MHT	244 (48.6)	1	229 (48.7)	14 (46.7)	186 (48.1)	53 (49.1)	205 (47.7)	38 (54.3)
**Tumor characteristics**								
Invasive tumor size								
>20 mm or skin and muscle involvement	127 (25.2)		120 (25.5)	6 (20.0)	98 (25.3)	28 (25.7)	104 (24.1)	22 (31.4)
Any axillary node involvement	189 (37.7)	2	178 (38.0)	11 (36.7)	142 (36.9)	43 (39.4)	155 (36.1)	34 (48.6)
Histological grade III	63 (12.5)		59 (12.5)	4 (13.3)	47 (12.1)	14 (12.8)	56 (13.0)	7 (10.0)
Hormone receptor status								
ER^+	503 (100)		471 (100)	30 (100)	378 (100)	109 (100)	431 (100)	70 (100)
PR^+	397 (78.9)		369 (78.3)	26 (86.7)	306 (79.1)	86 (78.9)	341 (79.1)	54 (77.1)
$HER2^+$	34 (7.5)	51	32 (7.6)	2 (7.4)	24 (7.1)	10 (9.8)	27 (7.0)	7 (11.7)
Triple negative	0 (0)							
**Final surgical technique**								
Mastectomy	206 (41.0)		191 (40.6)	14 (46.7)	162 (41.9)	42 (38.5)	177 (41.1)	28 (40.0)
**Adjuvant treatments**								
Radiotherapy	297 (59.0)		279 (59.2)	17 (56.7)	229 (59.2)	65 (59.6)	253 (58.7)	43 (61.4)
Chemotherapy	58 (11.5)		55 (11.7)	3 (10.0)	39 (10.1)	19 (17.4)	49 (11.4)	9 (12.0)
ER^+ only								
Ever tamoxifen	313 (62.2)		295 (62.6)	17 (56.7)	244 (63.0)	63 (57.8)	265 (61.5)	47 (67.1)
Ever aromatase inhibitor	206 (41.0)		198 (42.0)	8 (26.7)	159 (41.1)	43 (39.4)	172 (39.9)	34 (48.6)
**Type of event**								
Any breast cancer event	123 (24.5)		112 (23.8)	10 (33.3)	97 (25.1)	25 (22.9)	103 (23.9)	19 (27.1)
Any locoregional	42 (8.3)		40 (8.5)	2 (6.7)	34 (8.8)	7 (6.4)	35 (8.1)	7 (10.0)
Any contralateral	27 (5.4)		24 (5.1)	3 (10)	20 (5.2)	7 (6.4)	25 (5.8)	2 (2.9)
Any distant metastasis	78 (15.5)		69 (14.6)	8 (26.7)	61 (15.8)	16 (14.7)	63 (14.6)	14 (20.0)
Death due to any cause	112 (22.3)		99 (21.0)	12 (40.0)	89 (23.0)	21 (19.3)	92 (21.3)	19 (27.1)

Single nucleotide polymorphisms (SNPs), Body mass index (BMI), Menopausal hormone therapy (MHT), Estrogen receptor positive (ER^+), Progesterone receptor positive (PR^+), Human epidermal growth factor receptor 2 amplified ($HER2^+$)

Supplementary Table 4. Clinicopathological factors, treatments and breast cancer events in relation to AIB1 diplotypes in 503 patients with ER⁺ tumors

	**Patients with ER⁺ tumors**	Missing	**CGA_CGA**		**CGA_CCA**		**CGA_CGG**		**Rare**		Missing
			Yes	No	Yes	No	Yes	No	Yes	No	
	n = 503 (%)		n =296 (%)	n=198 (%)	n=88 (%)	n=406 (%)	n=58 (%)	n=436 (%)	n=52 (%)	n=442 (%)	n = 9 (%)
**Patients characteristics**											
Age ≥ 50 years	406 (80.7)		230 (77.7)	167 (84.3)	78 (88.6)	319 (78.6)	45 (77.6)	352 (80.7)	44 (84.6)	353 (79.9)	9 (100.0)
BMI ≥ 25 kg/m^2	227 (45.4)	3	121 (40.9)	97 (49.7)	45 (51.1)	173 (42.9)	33 (57.9)	185 (42.6)	19 (38.0)	199 (45.1)	9 (100.0)
Nulliparous	76 (15.1)		44 (14.9)	30 (15.2)	12 (13.6)	62 (15.3)	9 (15.5)	65 (14.9)	9 (17.3)	65 (14.7)	2 (22.2)
Current smoker	102 (20.3)		63 (21.3)	39 (19.7)	17 (19.3)	85 (20.9)	14 (24.1)	88 (20.2)	8 (15.4)	94 (21.3)	0 (0.0)
Coffee intake ≥ 2 cups/day	411 (81.7)		239 (80.7)	165 (83.3)	76 (86.4)	328 (80.8)	44 (75.9)	360 (82.6)	45 (86.5)	359 (81.2)	7 (77.8)
Alcohol abstainer	50 (9.9)		27 (9.1)	22 (11.1)	10 (11.4)	39 (9.6)	8 (13.8)	41 (9.4)	4 (7.7)	45 (10.2)	
Ever use of MHT	244 (48.6)	1	141 (47.6)	97 (49.2)	38 (43.7)	200 (49.3)	29 (50.0)	209 (48.0)	30 (57.7)	208 (47.2)	6 (66.7)
**Tumor characteristics**											
Invasive tumor size											
>20 mm or skin and muscle involvement	127 (25.2)		73 (24.7)	52 (26.3)	23 (26.1)	102 (25.1)	17 (29.3)	108 (24.8)	12 (23.1)	113 (25.6)	2 (22.2)
Any axillary node involvement	189 (37.7)	2	102 (34.7)	83 (41.9)	35 (39.8)	150 (37.1)	30 (51.7)	155 (35.7)	18 (34.6)	1067 (38.0)	4 (44.4)
Histological grade III	63 (12.5)		40 (13.5)	21 (10.6)	8 (9.1)	53 (13.1)	4 (6.9)	57 (13.1)	9 (17.3)	52 (11.8)	2 (22.2)
Hormone receptor status											
ER⁺	503 (100)		296 (100)	198 (100)	88 (100)	406 (100)	58 (100)	436 (100)	52 (100)	442 (100)	9 (100.0)
PR⁺	397 (78.9)		233 (78.7)	157 (79.3)	73 (83.0)	317 (78.1)	44 (75.9)	346 (79.4)	40 (76.9)	350 (79.2)	7 (77.8)
HER2⁺	34 (7.5)	51	16 (6.1)	18 (10.1)	7 (8.5)	27 (7.5)	6 (12.5)	28 (7.1)	5 (10.4)	29 (7.4)	0 (0)
Triple negative	0 (0)		0 (0.0)								0 (100)
**Final surgical technique**											
Mastectomy			124 (41.9)	79 (39.9)	34 (38.6)	169 (41.6)	24 (41.4)	179 (41.1)	21 (40.4)	182 (41.2)	0 (0.0)
**Adjuvant treatments**											
Radiotherapy	297 (59.0)		173 (58.4)	120 (60.6)	52 (59.1)	241 (59.4)	37 (63.8)	256 (58.7)	31 (59.6)	262 (59.3)	4 (44.4)
Chemotherapy	58 (11.5)		29 (9.8)	29 (14.6)	16 (18.2)	42 (10.3)	8 (13.8)	50 (11.5)	5 (9.6)	53 (12.0)	0 (0.0)
Endocrine therapy (ER⁺ only, n = 503)											
Ever tamoxifen	313 (62.2)		187 (63.2)	119 (60.2)	49 (55.7)	257 (63.3)	38 (65.5)	268 (61.5)	32 (61.5)	274 (62.0)	7 (77.8)
Ever AI	206 (41.0)		121 (40.9)	81 (40.9)	35 (39.8)	167 (41.1)	31 (53.4)	171 (39.2)	15 (28.8)	187 (42.3)	4 (44.4)
**Type of event**											
Any breast cancer event	123 (24.5)		68 (23.0)	228 (77.0)	22 (25.0)	66 (75.0)	16 (27.6)	42 (72.4)	15 (28.8)	37 (71.2)	2 (22.2)
Any locoregional	42 (8.3)		25 (8.4)	271 (91.6)	7 (8.0)	81 (92.0)	6 (10.3)	52 (89.7)	3 (5.8)	49 (94.2)	1 (11.1)
Any contralateral	27 (5.4)		15 (5.1)	281 (94.9)	6 (6.8)	82 (93.2)	1 (1.7)	57 (98.3)	5 (9.6)	47 (90.4)	0 (0)
Any distant metastasis	78 (15.5)		39 (13.2)	257 (86.8)	14 (15.9)	74 (84.1)	13 (22.4)	45 (77.6)	10 (19.2)	42 (80.8)	2 (22.2)
Death due to any cause	112 (22.3)		61 (20.6)	235 (79.4)	18 (20.5)	70 (79.5)	17 (29.3)	41 (70.7)	13 (25.0)	39 (75.0)	3 (33.3)

Body mass index (BMI), Menopausal hormone therapy (MHT), Estrogen receptor positive (ER⁺), Progesterone receptor positive (PR⁺), Human epidermal growth factor receptor 2 amplified (HER2⁺)

Supplementary Table 5. Adjusted HRs with 95% CI for all variables used in the multivariable model in 576 patients

	Interaction radiotherapy CGA_CGA			
		95% CI		
	HR	Lower	Upper	*P*-value
CGA_CGA	0.52	0.32	0.87	0.012
Interaction radiotherapy_CGA_CGA	2.13	1.06	4.28	0.033
Age ≥ 50 years	0.71	0.46	1.06	0.092
BMI ≥ 25 kg/m^2	1.45	1.02	2.05	0.037
Invasive tumor size >20mm or skin and muscle involvement	1.76	1.21	2.58	0.003
Any axillary node involvement	1.71	1.12	2.61	0.013
Histological grade III	1.53	0.95	2.45	0.079
ER^+	1.24	0.63	2.46	0.53
$HER2^+$	1.3	0.74	2.27	0.36
HER2 unknown	0.91	0.51	1.63	0.75
Radiotherapy	0.46	0.27	0.78	0.004
Chemotherapy	0.76	0.42	1.36	0.35
Tamoxifen	0.86	0.59	1.26	0.43
Aromatase inhibitor	0.67	0.43	1.05	0.78

Hazard ratio (HR), Confidence interval (CI), Body mass index (BMI), Estrogen receptor positive (ER^+), Human epidermal growth factor 2 amplified ($HER2^+$)

Supplementary Table 6. Adjusted HRs with 95% CI for all variables used in the multivariable model in 576 patients

	Interaction radiotherapy CGA_CCA			
	95% CI			
	HR	Lower	Upper	*P*-value
CGA_CCA	1.90	1.03	3.49	0.039
Interaction radiotherapy_CGA_CCA	0.31	0.12	0.81	0.017
Age ≥ 50 years	0.73	0.48	1.10	0.13
BMI ≥ 25 kg/m^2	1.46	1.03	2.07	0.033
Invasive tumor size >20mm or skin and muscle involvement	1.78	1.22	2.61	0.003
Any axillary node involvement	1.71	1.12	2.63	0.014
Histological grade III	1.54	0.96	2.48	0.073
ER^+	1.25	0.63	2.47	0.53
$HER2^+$	1.23	0.70	2.17	0.47
HER2 unknown	0.91	0.51	1.62	0.74
Radiotherapy	0.86	0.59	1.25	0.43
Chemotherapy	0.75	0.41	1.34	0.33
Tamoxifen	0.85	0.58	1.25	0.41
Aromatase inhibitor	0.67	0.43	1.05	0.079

Hazard ratio (HR), Confidence interval (CI), Body mass index (BMI), Estrogen receptor positive (ER^+), Human epidermal growth factor 2 amplified ($HER2^+$)

Supplementary Table 7. Adjusted HRs with 95% CI for all variables used in the multivariable model in 503 patients with ER+ tumors

	Interaction tamoxifen CGA_CGA			
		95% CI		
	HR	Lower	Upper	*P*-value
CGA_CGA	0.55	0.30	0.99	0.045
Interaction tamoxifen_CGA_CGA	1.99	0.92	4.30	0.079
Age ≥ 50 years	0.76	0.47	1.21	0.24
BMI ≥ 25 kg/m^2	1.58	1.08	2.31	0.017
Invasive tumor size >20mm or skin and muscle involvement	1.69	1.10	2.59	0.017
Any axillary node involvement	2.05	1.25	3.36	0.004
Histological grade III	1.84	1.09	3.09	0.022
HER2+	1.65	0.86	3.17	0.13
HER2 unknown	0.99	0.54	1.81	0.97
Radiotherapy	0.61	0.42	0.89	0.011
Chemotherapy	0.75	0.39	1.45	0.40
Tamoxifen	0.54	0.30	0.94	0.031
Aromatase inhibitor	0.49	0.30	0.81	0.005

Hazard ratio (HR), Confidence interval (CI), Body mass index (BMI), Human epidermal growth factor 2 amplified (HER2+)
